# NGF effects promote the maturation of rat pancreatic beta cells by regulating GLUT2 levels and distribution, and glucokinase activity

**DOI:** 10.1371/journal.pone.0303934

**Published:** 2024-06-14

**Authors:** Jazmín Samario-Román, Myrian Velasco, Carlos Larqué, René Cárdenas-Vázquez, Rosa Isela Ortiz-Huidobro, Marcia Hiriart

**Affiliations:** 1 Neuroscience Division, Cognitive Neuroscience Department, Instituto de Fisiología Celular, Universidad Nacional Autónoma de México, Mexico City, Mexico; 2 Department of Embryology and Genetics, Facultad de Medicina, Universidad Nacional Autónoma de México, Mexico City, Mexico; 3 Laboratory of Experimental Animal Biology, Facultad de Ciencias, Universidad Nacional Autónoma de México, Mexico City, Mexico; 4 Department of Genomic Medicine and Environmental Toxicology, Instituto de Investigaciones Biomédicas, Ciudad de México, Mexico; Nathan S Kline Institute, UNITED STATES

## Abstract

The nerve growth factor (NGF) participates in cell survival and glucose-stimulated insulin secretion (GSIS) processes in rat adult beta cells. GSIS is a complex process in which metabolic events and ionic channel activity are finely coupled. GLUT2 and glucokinase (GK) play central roles in GSIS by regulating the rate of the glycolytic pathway. The biphasic release of insulin upon glucose stimulation characterizes mature adult beta cells. On the other hand, beta cells obtained from neonatal, suckling, and weaning rats are considered immature because they secrete low levels of insulin and do not increase insulin secretion in response to high glucose. The weaning of rats (at postnatal day 20 in laboratory conditions) involves a dietary transition from maternal milk to standard chow. It is characterized by increased basal plasma glucose levels and insulin levels, which we consider physiological insulin resistance. On the other hand, we have observed that incubating rat beta cells with NGF increases GSIS by increasing calcium currents in neonatal cells. In this work, we studied the effects of NGF on the regulation of cellular distribution and activity of GLUT2 and GK to explore its potential role in the maturation of GSIS in beta cells from P20 rats. Pancreatic islet cells from both adult and P20 rats were isolated and incubated with 5.6 mM or 15.6 mM glucose with and without NGF for 4 hours. Specific immunofluorescence assays were conducted following the incubation period to detect insulin and GLUT2. Additionally, we measured glucose uptake, glucokinase activity, and insulin secretion assays at 5.6 mM or 15.6 mM glucose concentrations.

We observed an age-dependent variation in the distribution of GLUT2 in pancreatic beta cells and found that glucose plays a regulatory role in GLUT2 distribution independently of age. Moreover, NGF increases GLUT2 abundance, glucose uptake, and GSIS in P20 beta cells and GK activity in adult beta cells.

Our results suggest that besides increasing calcium currents, NGF regulates metabolic components of the GSIS, thereby contributing to the maturation process of pancreatic beta cells.

## Introduction

Nerve growth factor (NGF) is a pleiotropic neurotrophin, synthesized by neurons and non-neural cells. It plays a crucial role in the maturation of various organs and participates in the regulation of metabolic homeostasis of the body [1]. Pancreatic beta cells from humans and rodents synthesize and secrete NGF [2]. Furthermore, these cells express the high-affinity TrkA receptors, as well as the low-affinity p75^NTR^ neurotrophin receptors, (member of the tumor necrosis factor receptor TNFR superfamily) receptors, for NGF [3, 4].

NGF participates in cell survival and glucose-stimulated insulin secretion (GSIS) [1, 5]. In mature beta cells, GSIS is a finely regulated mechanism where the glucose entry rate, phosphorylation, and catabolic processing couple the extracellular glucose concentrations and the pancreatic beta-cell secretory activity. Multiple factors regulate insulin secretion; however, glucose is the main secretagogue [6]. The rise of extracellular glucose concentrations increases glucose uptake through GLUT2, the glucotransporter with the highest Km [7], which plays a critical role in GSIS in rodent beta cells [8]. After internalization, glucose is immediately phosphorylated by glucokinase and metabolized in glycolysis. This metabolic pathway generates pyruvate, which enters the Krebs cycle within the mitochondria, increasing the ATP/ADP relation that induces the closure of ATP-sensitive potassium channels, which triggers the depolarization of the membrane and the biphasic secretion of insulin granules [9].

On the other hand, cells that express GLUT2 and the high Km enzyme glucokinase (GK), which is considered the limiting factor in determining the glycolytic rate [10]. Thus, reducing GLUT2 levels at the cell membrane may decrease glucose availability for glucokinase. This phenomenon has been observed in pathological processes, such as diabetes, and in physiological conditions during the early stages of postnatal development in rodents [8, 11]. Pancreatic beta cells obtained from lactating rats do not exhibit a biphasic insulin secretory response to increasing extracellular glucose concentrations [12]. In addition, glucokinase gene expression may be related to the maturation of the secretory function of pancreatic beta cells [13]. Pancreatic beta cells possess an inactive form of glucokinase located at the membrane of the secretory insulin vesicles [14]. After glucose internalization to the cells, glucokinase undergoes a conformational change induced by PFK2/FBPase-2 that releases its active form into the cytoplasm [14, 15]. The loss of GSIS in rodent beta cells is related to decreased levels in the active form of GK [11, 16, 17]. Furthermore, neonatal rats’ beta cells show lower GK levels than adult beta cells [11, 18].

As mentioned earlier, beta cells of neonatal rats are immature due to the lack of insulin secretory response to increasing glucose levels. Around postnatal day 28, pancreatic beta cells begin to show a biphasic insulin secretion similar to that observed in adult beta cells [19]. Therefore, adult beta cells are mature due to their capability to secrete a robust insulin response to increased extracellular glucose concentrations [19]. The mechanisms underlying the maturation process of beta-cell GSIS are partially understood. However, several studies have reported quantitative and functional changes in the electrical components involved in this process during early postnatal development [12, 20].

Previous work conducted by our group described that islet cells reorganize during weaning [19, 21, 22]. In addition, P20 rats exhibit a physiological condition of insulin resistance characterized by high plasma levels of glucose and insulin [19]. Furthermore, in adult rats NGF produced and secreted by beta cells promotes cell survival and also enhances insulin secretion by increasing Na^+^ and Ca^2+^ current densities [2, 21, 22]. NGF may participate in the maturation of GSIS due to its modulating effects on neonatal beta-cell Ca^2+^ current density and synthesis of Ca^2+^ channels observed after incubation with this neurotrophin [23]. These works suggest that NGF could play a role in the functional maturation of beta cells by regulating the electrical components of the GSIS. On the other hand, we previously analyzed a transcriptome focused on beta-cell maturation in P20 and adult beta cells [24]. These studies revealed that adult beta cells expressed more Slc2a2 (encoding gene for GLUT2) than the P20 beta cells [24]. Moreover, a complementary regulatory network analysis demonstrated a significant probabilistic dependence relationship between GLUT2 and NGF expression (unpublished data).

Functional maturation is the process by which pancreatic beta cells develop a robust secretory response. Multiple factors, including hormones, glucose, other nutrients, and the dietary transition during weaning are considered promoters of functional maturation, with the dietary transition being the most relevant factor [24, 25]. During the weaning period, the transition from maternal milk, which is rich in fatty acids, to a carbohydrate-predominant diet triggers the adaptation of the metabolic machinery enabling beta cells to produce high levels of ATP through glucose metabolism [25].

The increase in expression, protein levels, extracellular glucose-sensing, and glucose-phosphorylase activity of GLUT2 and GK constitute crucial elements of the functional maturation of pancreatic beta cells [26]. As mentioned, NGF increases GSIS in neonatal beta cells by increasing calcium current density [23]. Therefore, NGF may promote beta-cell functional maturation. However, to our knowledge, no studies have assessed the NGF effects on GSIS in beta cells obtained from P20 rats, characterized by physiological insulin resistance due to high fasting plasma glucose and insulin levels [19, 27].

We hypothesized that NGF may contribute to the functional maturation of beta cells by regulating critical metabolic components of the GSIS. Therefore, this study aimed to assess the effects of NGF on the protein levels and distribution of GLUT2, and the activity of GK and GSIS in islet cells obtained from P20 and adult rats.

Our results exhibit that NGF, and glucose modulate the subcellular distribution of GLUT2 in beta cells obtained from P20 and adult rats. Additionally, our work contributes to elucidating the mechanisms through which NGF regulates the maturation of pancreatic beta cells.

## Materials and methods

### Experimental animals

All the methods used in this study were approved by the animal care committee of the Instituto de Fisiología Celular in the Universidad Nacional Autónoma de México. The care of the animals was carried out following the guidelines of international standards for Biomedical research of Animals, Council of International Organizations of Medical Sciences, 2010. Male Wistar rats were used for all experiments. Newly weaned 20 days postnatal (P20) and young adult 250–280 g, approximately 8 weeks of age Wistar rats were used. All animals were kept in the local animal facility and kept in a 12:12 h light: dark cycle. For each analysis, ‘n’ represents a batch of 2 adult rats and 6 P20 rats.

### Primary culture of pancreatic islet cells

Before each experiment, adult rats were fasted for 12 h while P20 rats were fasted for 4 h. These fasting times were used because glucose reached basal levels and remained stable after this fasting period for each age [27]. Fasted animals were anesthetized with sodium Pentobarbital (40 mg/kg of weight) intraperitoneally; after the dissection, cervical dislocation was performed to sacrifice them. Pancreatic islet cells from P20 and adult rats were obtained following the previously described methodology [28]. Briefly, pancreases were fragmented and digested with collagenase IV (0.3 mg/ml) (for 5 min at 37° C in a shaking bath). Once a homogeneous mixture was obtained, cold Hank’s solution was added. The resulting solution/mixture was centrifuged at 185 g for 3 minutes, the supernatant was removed, and Hank’s solution was added and repeated three times.

Islets were collected manually. Subsequently, a dispersant solution was added (calcium-free Spinner solution, with glucose 15.6 mM, 0.5% BSA, and 0.01% trypsin). Islets were stirred for 3 minutes at 37° C and then mechanically dispersed using a pipette. Cell counting was performed by trypan blue dye staining in a Neubauer chamber. Before the experiments, they were kept overnight in an RPMI medium containing 10% FBS (fetal bovine serum) and 11.1 mM glucose.

### Immunofluorescence

**Fig 1:**
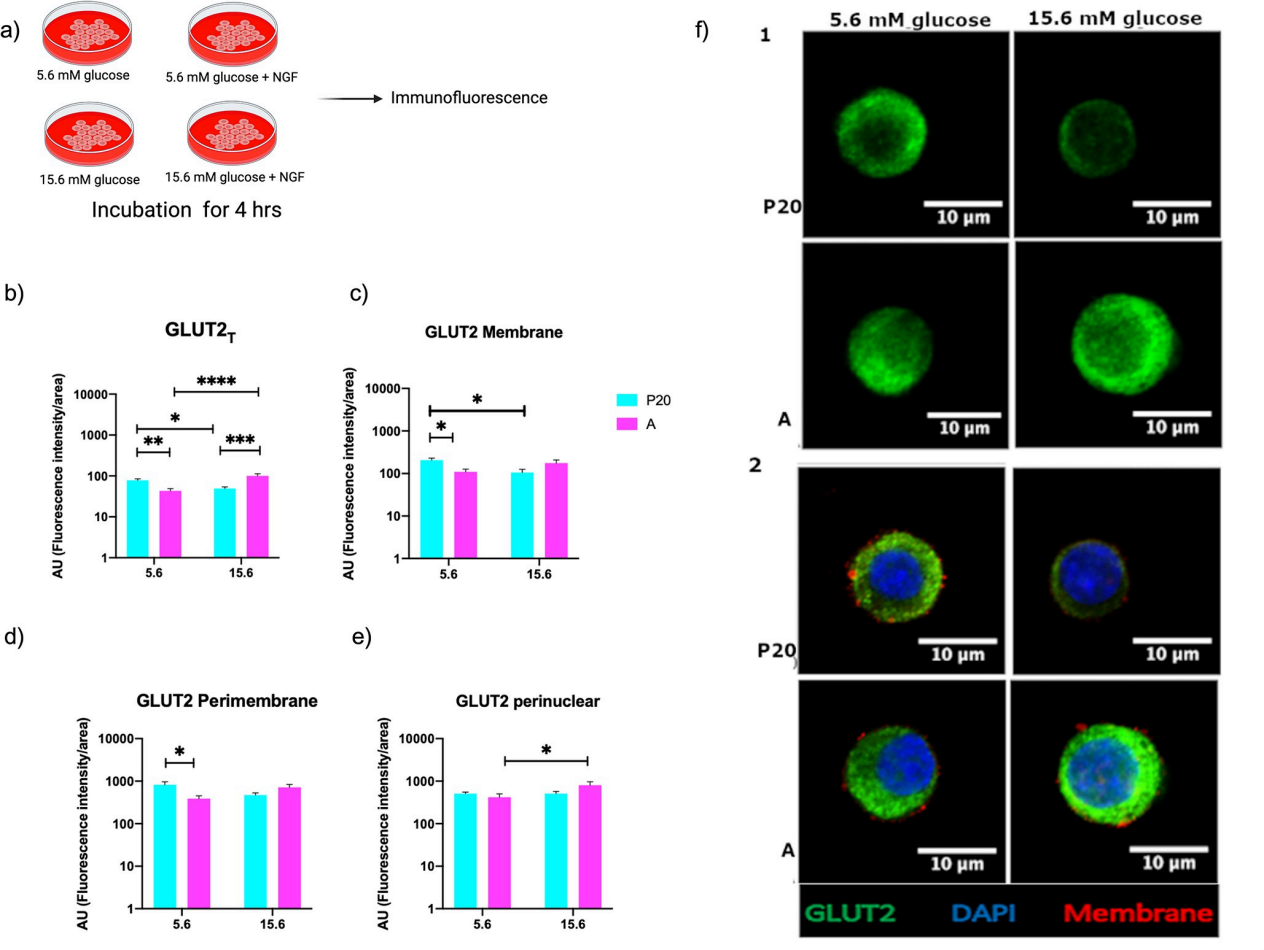
GLUT2 distribution in pancreatic beta cells. Pancreatic islet cells obtained from P20 and adult rats were incubated at low (5.6mM) and high (15.6 mM) glucose concentrations. (a) Experimental design. Fluorescence levels of GLUT2 normalized to the cell area, in the total cell area (GLUT2_T_). (b); in the membrane (GLUT2_M_) (c); in the peri-membranal zone (GLUT2_PM_) (d); and perinuclear zone (GLUT2_PN_)(e) of P20 and A beta cells. (f) Representative confocal micrographs of P20 and A beta cells incubated in 5.6 mM glucose (left) and 15.6 mM (right). 1: GLUT2 total, 2: membranal. Green = GLUT2, blue = nucleus, red = membrane. Bars represent the mean ± S.E.M. * p < 0.05, ** p < 0.005, two-way analysis of variance ANOVA test. # p < 0.05, t test.

Islet cells were seeded on coverslips previously treated with poly-l-lysine and left overnight in RPMI, 10% FBS. The following day the culture medium was changed to RPMI 1% FBS with 5.6 mM or 15.6 mM glucose concentration, and 50 ng/dl of NGF 2.5S only to the treated groups [3, 22, 23]. Pancreatic islet cells were incubated for 5 minutes, 1 hour, and 4 hours for the first series of experiments to determine the effects of different incubation times with NGF over the GLUT2 levels (S1 Fig). The analysis of immunofluorescence assays showed that after 4 h of incubation with NGF, the GLUT2 levels in beta cells increased when compared to control (S1 Fig). Therefore, the following experiments were performed after 4 hours of incubation with NGF. Similarly, incubation with different glucose concentrations (5.6 mM or 15.6 mM) for 4 hours was used to assess its effects on GLUT2 levels and subcellular localization in beta cells obtained from P20 and adult rats [29] (Fig 1).

Finally, pancreatic islet cells were fixed with paraformaldehyde 4% for 30 minutes. They were kept for 15 minutes in perforating/blocking solution (PBS, goat serum 1%, and triton X-100 0.1%). Cells were incubated overnight with insulin-antibody 1:5000 (raised in guinea pig) at 4° C overnight. The next day, primary antibody was rinsed and secondary antibody FIT-C 1: 100 (anti guinea pig) was added and incubated for one hour. After this, anti guinea pig secondary antibody was washed, and GLUT2 1: 200 (ABCAM) antibody was added and left overnight. The next day, Alexa 649 secondary antibodies 1: 100 (anti-rabbit) were added and incubated for one hour. The samples immuno-stained with GLUT2 antibody were rinsed and incubated overnight with ATP1A1 1: 100 antibody (Thermo Fisher Scientific), as a membrane marker. The secondary antibody Alexa 555 1: 100 (anti-mouse) was added, incubating for one hour. DAPI 1: 100. They were finally mounted in medium with 15 mM NaN3 (DAKO).

### Epifluorescence and confocal microscopy and fluorescence quantification on pancreatic beta cells

The micrographs were taken in an epifluorescent Olympus IX71 inverted microscope, with a mercury lamp and 651 nm-emission 667 nm excitation filter for Alexa Fluor 647 and the 488 nm-522 nm emission excitation filter for FITC. Images were captured separately using a 20x objective with the Q Imagin digital camera, ImagePro DS 6.0 software.

The acquisition of image stacks (z-stack) by confocal microscopy was performed with the Zeiss LSM800 inverted confocal microscope using lasers of 405, 488, 546, and 647 nm, with a sequential configuration of channels to avoid “crosstalk.” The microscope operates with the ZEN blue version 2.6 software.

Images were processed and analyzed with ImageJ 1.36 (Wayne Rasband; National Institutes of Health, USA. Background-corrected fluorescence levels were calculated for each sample and the negative controls. Data were collected from three independent experiments with at least ten cells from each assay (three cultures of three rats each for each age). Analysis of the specific fluorescence levels of GLUT2 and insulin; as well as the fluorescence by zones of GLUT2 located in the membrane (M), close to the membrane (membrane periphery: PM), and close to the nucleus (perinuclear: PN) of confocal images was carried out with free software Fiji (ImageJ). The analyzed area was selected for each cell. The mean fluorescence levels were obtained by Stack. Background-corrected fluorescence levels was calculated for each sample, and the negative controls; fluorescence levels were normalized to cell area. The image stacks corresponding to the middle zone of the cell (approximately half of the stacks) were chosen, a Z-projection was made, and a mixture of the channels corresponding to the membrane marker, GLUT2, and nucleus was made.

### Protein extraction and Western blot

Islet protein extracts were obtained by homogenization using RIPA lysis buffer. Total protein was quantified by Bradford´s method. The protein extracts were separated by SDS-PAGE in 8% polyacrylamide gels and transferred to a PVDF membrane using an accelerated semi-dry blotting transfer system (Trans-blot turbo, Bio-Rad Laboratories, Hercules CA, USA). The membranes were blocked with non-fat dry milk 5% in TBS-tween 1% for 1h at room temperature and incubated overnight at 4°C with rabbit polyclonal anti-glucose transporter GLUT2 (Ab54460) diluted 1:500 in blocking solution. Goat polyclonal anti-β-actin (SCB#sc1616) diluted 1:5000 in blocking solution were used for the detection of load control protein. After incubation with secondary antibodies, proteins were visualized using a chemiluminescent HRP substrate method with Immobilon Western Chemiluminescent HRP substrate (#WBKLSO100, Merck Millipore, Mass, USA). Densitometric analysis was performed using ImageJ software (NHI 1.44) and represented by the mean intensity of bands obtained from three independent experiments.

### Glucose uptake

The pancreatic islet cells were seeded in 96-well plates for fluorescence detection (50,000 pancreatic islet cells in the case of adults and 80,000 pancreatic islet cells for P20) each group in duplicate. Incubations were carried out in different conditions (50 ng/mL of NGF 2.5S and 5.6 mM or 15.6 mM of glucose, and control) for 4 hours. A labeled glucose solution (2-NBDG-Cell-Based Assay NBD-Glucose Cayman’s Glucose Uptake Cell-Based Assay Kit / Item No. 600470) prepared at two different glucose concentrations: 5.6 mM and 15.6 mM in Hank’s solution without glucose. Once the incubation time had elapsed, the media were removed, and 100 μl Hank’s assay solution (2.8 mM glucose concentration) was added to each well and kept at 37°C and 5% CO_2_ for 30 minutes. After that, the medium was removed, and 100 μl of Hank’s solution without glucose was added to each well, and 50 μl of the 2-NBDG 3X solution was added to each well, depending on the glucose concentration. Glucose internalization was registered for 10 minutes on a Flex Station 3 equipment. The glucose uptake efficiency was calculated using the fluorescence intensity registered during 10 minutes normalized by the number of cells (Fluorescence/min/cell). The data were from 3 independent experiments (3 cultures).

### Flow cytometry

The pancreatic islet cells were incubated in microtubes for 4 hours in RPMI containing 1% FBS with 5.6 mM glucose concentration, or 15.6 mM glucose concentrations with 50 ng/ml of NGF 2.5S for the treated group. The labelled glucose solution (2-NBDG) was prepared at two different glucose concentrations: 5.6 mM and 15.6 mM. Hank’s 2.8 mM glucose solution was added to each tube, and they were kept for 30 minutes at 37°C and 5% CO_2_. The medium was removed, 300 μl of 2-NBDG solution were added to each tube, (depending on the glucose condition), and samples were analyzed for 10 minutes. For the identification of the beta-cell subpopulations, the previously reported standardization was followed [24]. During cytometric analysis, live beta cells were identified and classified either in low glucose uptake (Low uptake) or high glucose uptake (High uptake) cells. (Fig 3B). The data were from 3 independent experiments.

### Glucokinase activity

For this assay, 800,000 pancreatic islet cells were used for the P20 groups and 400,000 pancreatic islet cells for the adult groups. Cells were incubated in microtubes at 37°C and 5% CO_2_, for 4 hours in 1% RPMI 5.6 mM and 15.6 mM glucose plus 50 ng/ml NGF 2.5S for the treated group. After incubation, the medium was removed and 200 μl of KH_2_PO_4_, KCl, MgCL_2_, EDTA, DTT buffer were added to each tube. Tube content was gently homogenized with using a manual homogenizer for approximately 7 minutes keeping it on ice, then it was centrifuged at 10,000 g for 15 minutes at 4°C. The supernatant which contained the cytoplasmic proteins was separated.

20 μl of cell supernatant, 110 μl of HEPES buffer (1M, pH 7.5), 100 μl of reaction cocktail (Tris HCl buffer, pH 9, MgCl2, ATP, glucose, ß-NADP) were placed in a 96-well plate and incubated at 30°C, pH 9 and 1 cm of light path for 10 minutes. Reading was performed with 340 nm laser at 30°C for 10 minutes. The cells were washed three times with phosphate buffer (PBS) and lysed with RIPA buffer containing a protease inhibitor, and the protein was quantified by the Bradford method. Normalization and calculation were carried out with the following formula: Activity: [(ADO / min) / ƐuM x (Vol. assay / 1000) x dilution x1 / Vol. assay enzyme] where ADO/min = slope of readings. The data were from 3 independent experiments (3 different cultures).

### Insulin secretion assay

The pancreatic islet cells were seeded in 24-well plates (50,000 pancreatic islet cells for adults and 80,000 pancreatic islet cells for P20) each group in duplicate. Incubations were carried out in the different treatments (5.6 mM and 15.6 mM of glucose control and with 50 ng/ml of NGF) for 4 hours. After the incubation, the media were removed, and Hank’s assay solution (2.8 mM glucose) was added and kept at 37°C and 5% CO_2_, for 30 minutes. After time elapsed, the medium was removed, and 500 μl of Hank’s assay solution with 5.6 mM glucose, was added to the basal condition wells, and 500 μl of Hank’s assay solution with 15.6 mM glucose was added to the wells of the stimulated condition, and kept for 1 hour at 37°C and 5% CO_2_. After incubation, the supernatants were collected and quantified using Ultrasensitive rat insulin ELISA system according to manufacturer’s instruction (Mercodia Uppsala, Sweden). The insulin concentrations were normalized with the number of cells that were seeded in each well for each age.

### Statical analysis

All analyses were conducted using GraphPad Prism and Microsoft Excel. The data are reported as the mean ± standard-error of the mean (S.E.M.). Statistical significance was considered for p values < 0.05. The statical analysis, t-test were performed, two-way analysis of variance ANOVA and analysis of variance with post hoc Tukey’s tests were performed.

## Results

### Effect of different glucose concentrations on levels and the subcellular distribution of GLUT2

To determine whether glucose regulates the subcellular distribution of GLUT2, we analyzed three subcellular areas in beta cells (ins^+^), the cell membrane area (GLUT2_M_), the contiguous area of the membrane, which we called peri-membranal (GLUT2_PM_), and the area that surrounds the nucleus, perinuclear (GLUT2_PN_)_._ Adult beta cells exhibited increased levels GLUT2_T_ and GLUT2_PN_ at high-glucose concentrations compared to low glucose concentrations (Fig 1B, 1E and 1F). Conversely, P20 beta cells displayed higher levels of GLUT2_T,_ and GLUT2_M_ at 5.6 mM of glucose than when incubated at higher glucose levels (Fig 1B and 1C). On the other hand, P20 beta cells also showed higher levels of GLUT2_T_, GLUT2_M_, and GLUT2_PM_ than adult beta cells after incubation with 5.6 mM of glucose (Fig 1B–1D).

### Effect of NGF on GLUT2 levels

**Fig 2:**
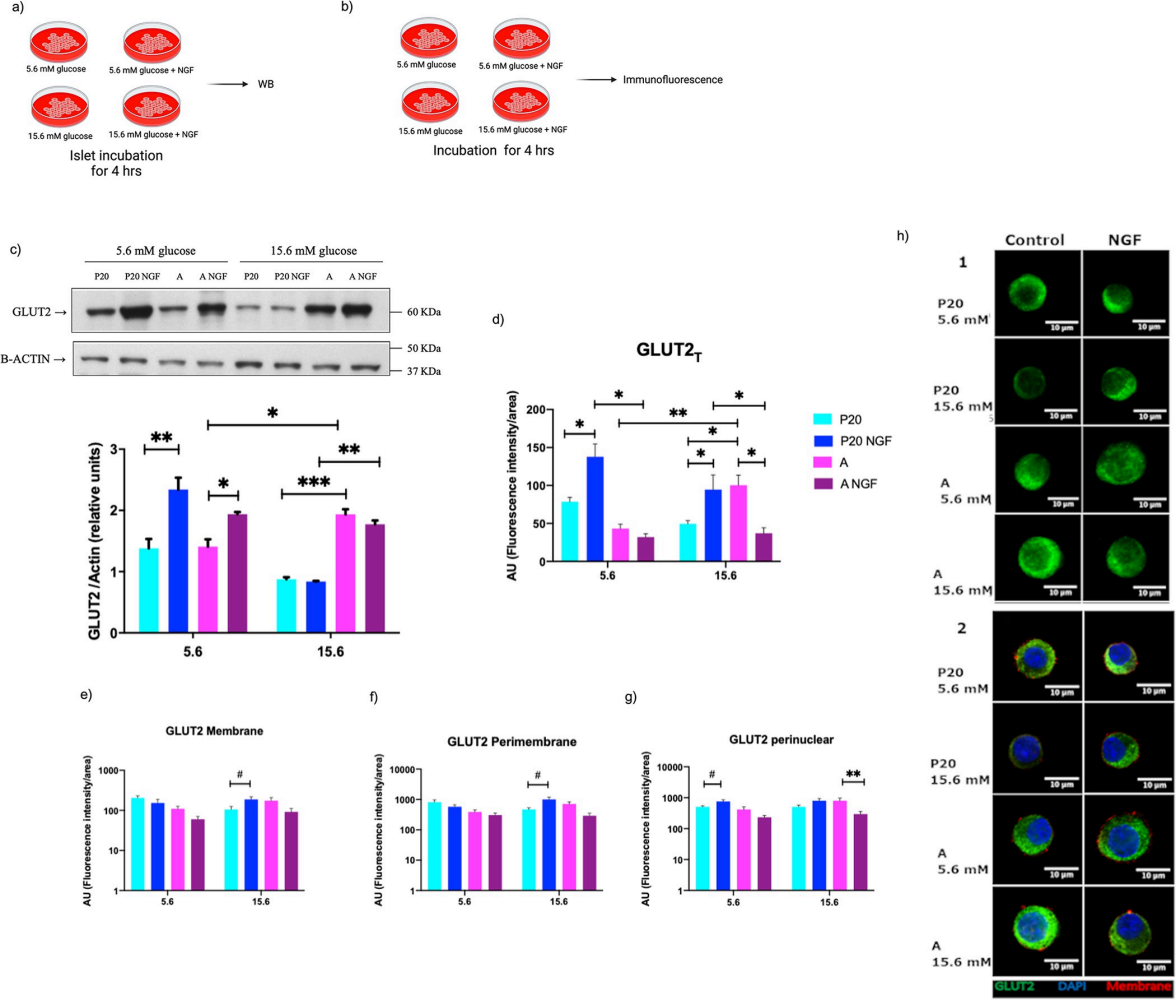
NGF effects on GLUT2 levels in pancreatic islets and GLUT2 distribution in beta cells. (a)(b) Experimental design. (c) Western blot analysis of protein extracts separated by SDS-PAGE of pancreatic islets obtained from P20 and adult rats, incubated with or without 50 ng/mL of NGF, at low (5.6 mM) or high (15.6 mM) glucose conditions. Semiquantitative analysis of fluorescence intensity to determine GLUT2 abundance in the total cell area (GLUT2_T_) (d) in the membrane (GLUT2_M_) (e) in the peri-membranal zone (GLUT2_PM_) (f) and in the perinuclear zone (GLUT2_PN_) (g) in beta cells obtained from P20 and adult rats incubated with or without 50 ng/mL of NGF, at low (5.6 mM) or high (15.6 mM) glucose concentrations. (h) Representative confocal micrographs of fluorescent specific- immunoreactivity to detect GLUT2 in beta cells obtained from P20 and adult rats incubated with or without 50 ng/mL of NGF, at low (5.6 mM) or high (15.6 mM) glucose concentrations. Bars represent the mean ± S.E.M. * p <0.05, ** p <0.005, two-way analysis of variance ANOVA test. # p<0.05, t test.

Pancreatic islets obtained from P20 and adult rats were incubated at different concentrations of glucose with or without NGF. We observed that NGF caused an increase in GLUT2 protein abundance in P20 and adult islets when incubated at 5.6 mM glucose compared to control conditions (Fig 2C). In addition, NGF also increases GLUT2_T_, and GLUT2_PN_ fluorescence levels in P20 beta cells at 5.6 mM glucose when compared to control conditions (Fig 2D, 2G and 2H). Moreover, NGF increased GLUT2_T_, GLUT2_M_, and GLUT2_PM_ levels in P20 beta cells incubated at 15.6 mM compared with incubation at 15.6 mM alone (Fig 2D–2F). However, incubation of adult beta cells in 15.6 mM glucose and NGF caused a decrease in GLUT2_T_, and GLUT2_PN_ levels when compared to the respective control (Fig 2D and 2G).

### Glucose uptake in P20 and adult pancreatic islet cells

**Fig 3:**
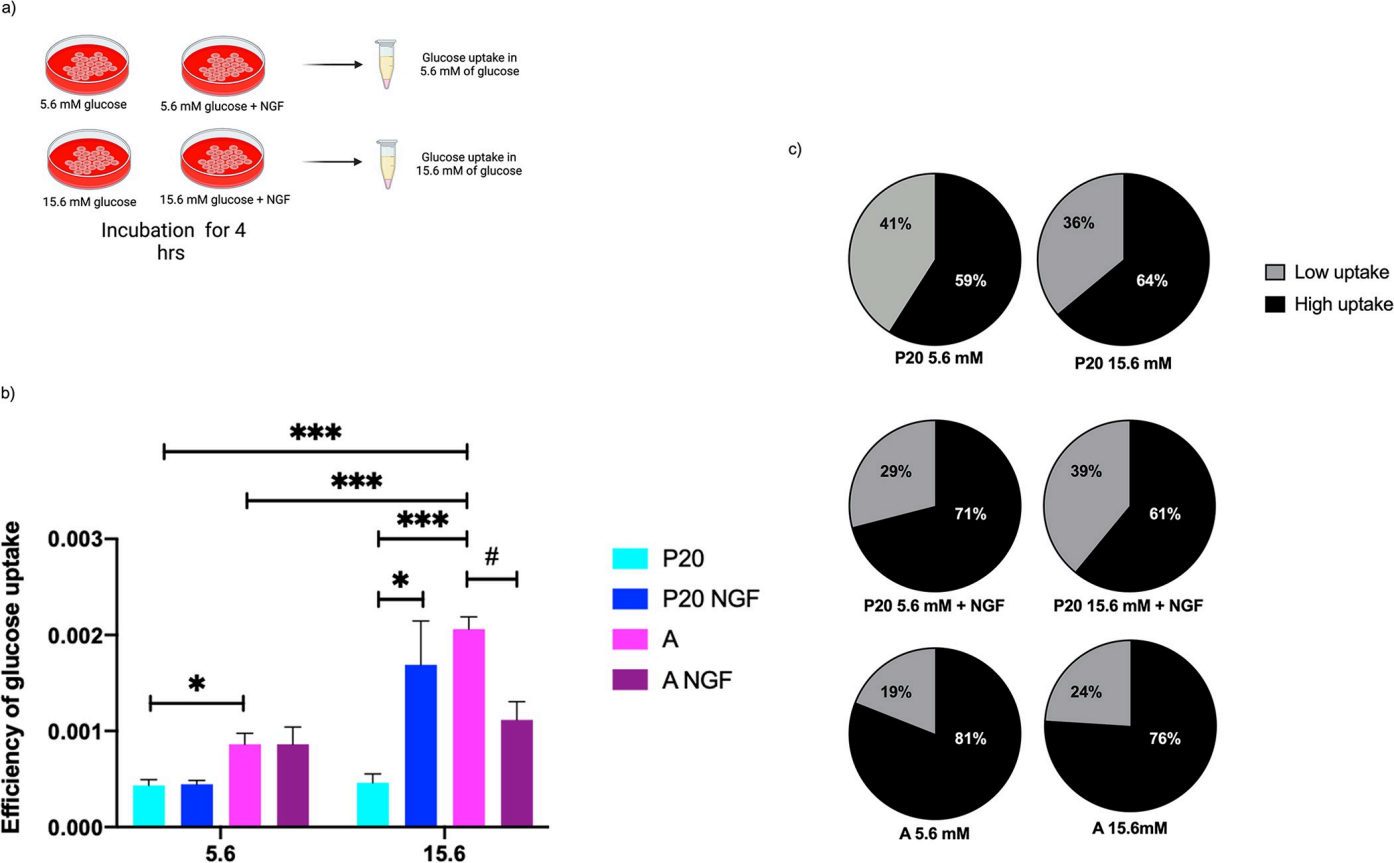
Analysis of glucose uptake by P20 and adult beta cells. (a) Experimental design. (b) Glucose uptake efficiency (expressed as increase of fluorescence levels per cell per minute) of P20 and adult (A) beta cells exposed to 50 ng/mL of NGF at 5.6 mM or 15.6 mM glucose levels, and control. (c) Proportion of beta-cell subpopulations with low and high glucose uptake analyzed by flow cytometry. Bars represent the mean ± S.E.M. * p < 0.05, ** p < 0.005, *** p < 0.000, two-way analysis of variance ANOVA test, # p < 0.05, t test.

To determine the functional role of GLUT2 levels and distribution, we analyzed glucose uptake assays using 2-NBDG-glucose. P20 pancreatic islet cells exhibited a lower glucose uptake efficiency (see materials and methods) in both glucose concentrations compared to adult pancreatic islet cells (Fig 3B). NGF increased the glucose uptake efficiency of P20 beta cells only when exposed to 15.6 mM glucose concentrations compared to control. On the contrary, NGF decreased the glucose uptake efficiency of adult beta cells incubated at 15.6 mM glucose concentrations compared to control.

A flow cytometry assay was performed to further explore beta-cell glucose uptake (Fig 3C). Interestingly, low-uptake (L) and high-uptake (H) beta-cell subpopulations were identified at both 5.6 mM and 15.6 mM glucose conditions. An age-dependent increase in the proportion of H beta cells was observed at 5.6 mM glucose concentrations. In addition, incubation of P20 cells with NGF induced an increase in the proportion of H beta cells observed at 5.6 mM glucose conditions.

### Glucokinase activity in P20 and adult pancreatic islet cells

**Fig 4:**
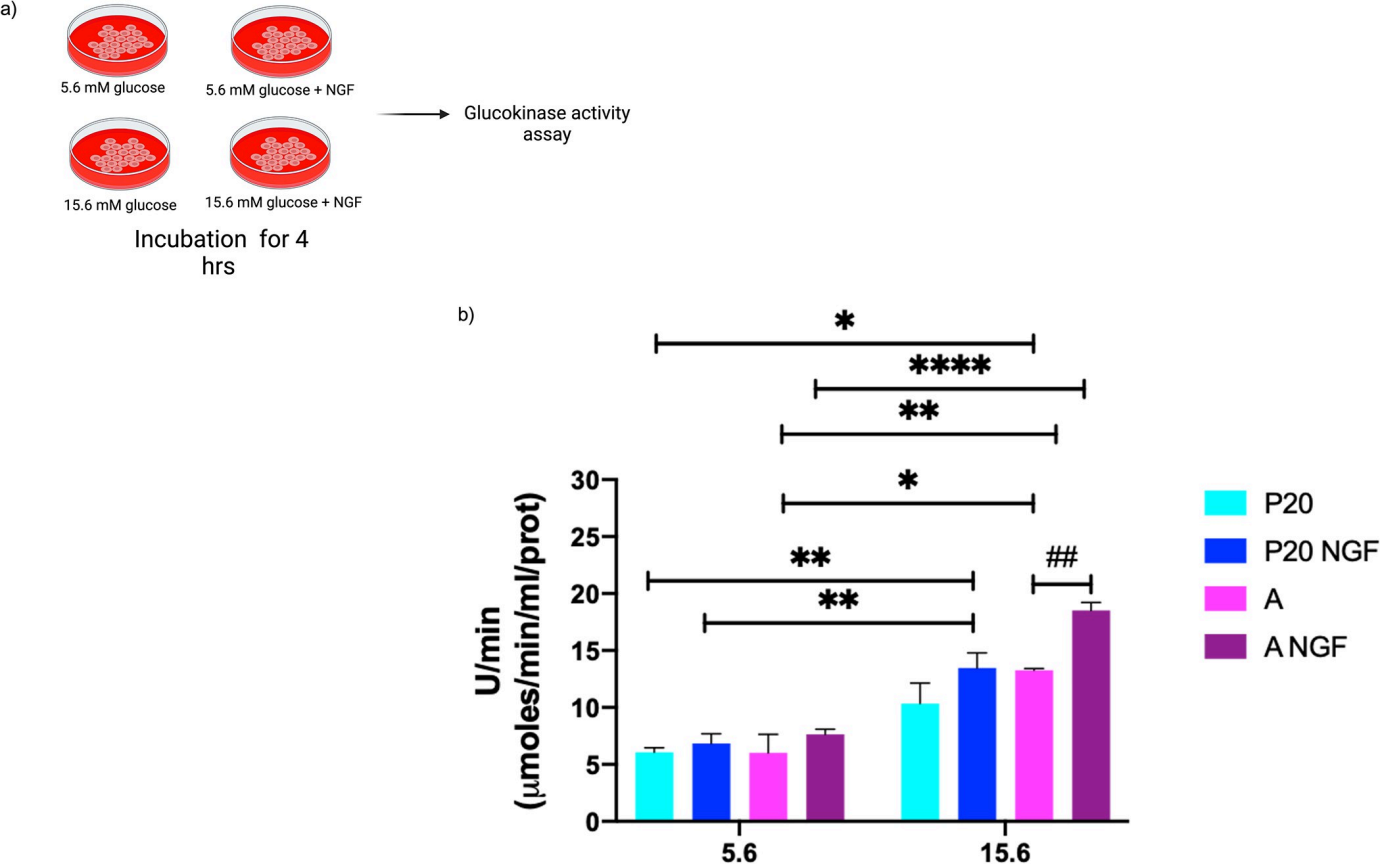
Glucokinase activity in P20 and adult beta-cells. (a) Experimental design. (b) Glucokinase activity in P20 and adult (A) pancreatic islet cells exposed to 50 ng/mL of NGF at low (5.6 mM) or high (15.6 mM) glucose levels, and control. Bars represent the mean ± S.E.M. * p < 0.05, ** p < 0.005, *** p < 0.0005, two-way analysis of variance ANOVA test.

Since glucokinase (GK) determines the glycolytic rate after glucose internalization, we measured GK activity. As shown in Fig 4B, the enzyme activity of GK in P20 and adult pancreatic islet cells was higher after a 4-hour incubation at 15.6 mM glucose than in 5.6 mM glucose concentrations. In addition, an increased GK activity in adult islet cells was observed after incubation with NGF at 15.6 mM glucose concentrations compared to control.

No difference in GK activity was observed in adult islet cells after incubation with NGF at 5.6 mM glucose concentrations. Moreover, we did not observe the effects of NGF on GK activity in P20 islet cells at either glucose concentration.

### Effects of preincubation with NGF and different glucose concentrations on pancreatic beta cells insulin abundance and glucose-stimulated insulin secretion

**Fig 5:**
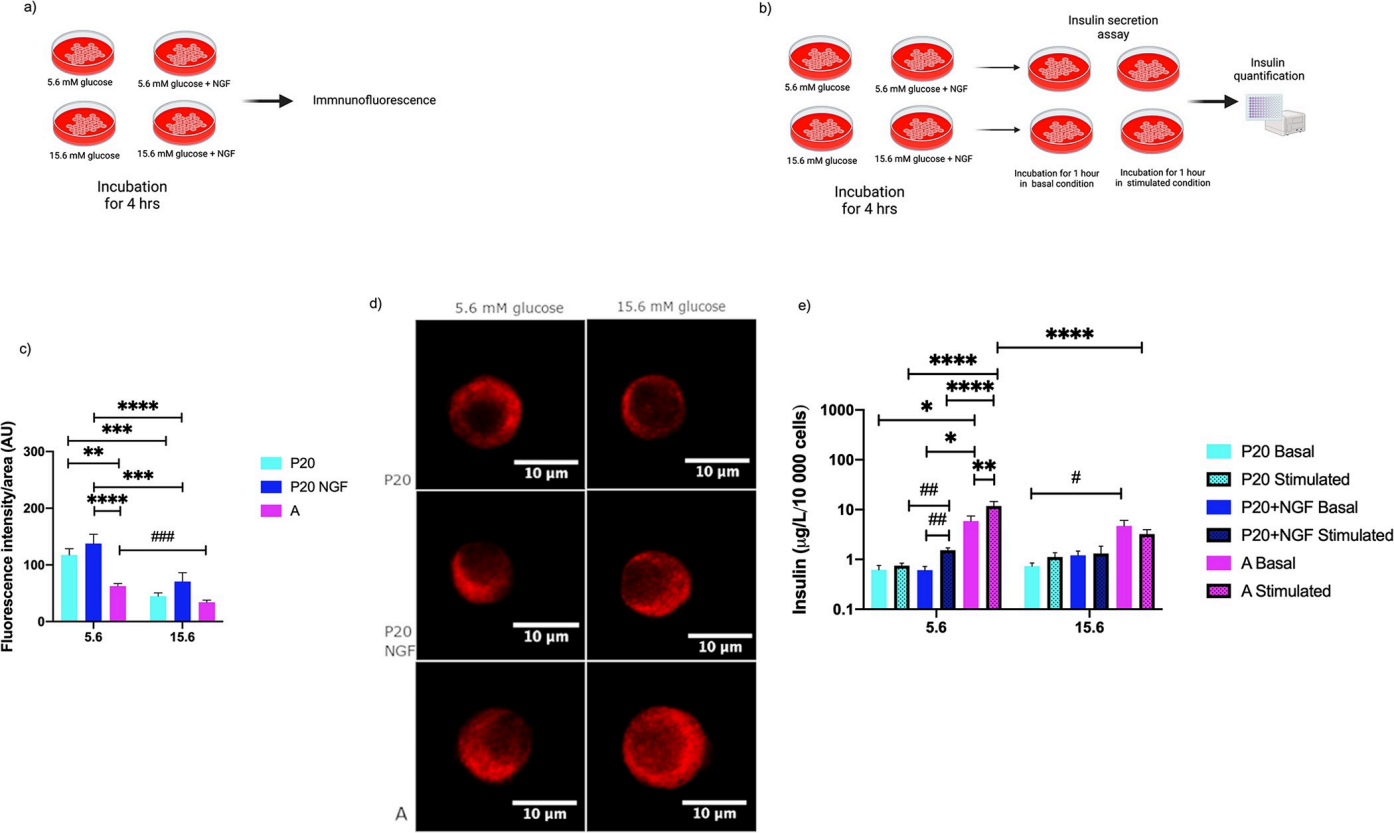
Insulin levels, and glucose-stimulated insulin secretion of beta cells obtained from P20 and adult rats, after preincubation with NGF at 5.6 mM or 15.6 mM glucose, as well as under control conditions. (a) (b) Experimental design. (c) Analysis of intracellular insulin levels (determined by quantification of fluorescence intensity after insulin immunodetection) of P20 and adult (A) beta cells after a 4-h preincubation at 5.6 mM and 15.6 mM glucose concentrations. (d) Representative confocal micrographs of P20 beta cells after incubation at 5.6 mM and 15.6 mM glucose concentrations with or without NGF (50 ng/mL), and of after incubation at low and high glucose concentrations Red = insulin. (e) Insulin secretion at basal (5.6 mM) and stimulating (15.6 mM) glucose concentrations assessed in P20 beta cells after a 4-h preincubation at low and high glucose concentrations with or without NGF (50 ng/mL); and in beta cells after a 4-h preincubation at low and high glucose conditions. Bars represent the mean ± S.E.M. * p < 0.05, ** p < 0.005, *** p < 0.0005, **** p < 0.0001, two-way analysis of variance ANOVA test, ## p < 0.005 t test.

We explored the effects of preincubation with NGF and different glucose concentrations on beta-cell insulin abundance to assess the potential impact of extracellular glucose levels and NGF on the maturation process of beta cells (Fig 5C and 5D). P20 and adult beta cells exhibited higher insulin levels after a 4-hour preincubation at 5.6 mM glucose than preincubation at 15.6 mM glucose concentration (Fig 5C and 5D). Moreover, P20 beta cells showed higher levels of intracellular insulin compared to adult beta cells after a 4-hour incubation at 5.6 mM glucose concentrations. However, NGF at 5.6 mM glucose affected P20 beta-cell insulin levels after a 4-hour preincubation, compared to the control.

After being preincubated with NGF at 5.6 mM or 15.6 mM glucose concentrations, pancreatic islet cells obtained from P20, and adult rats were subsequently subjected to an insulin secretion assay.

Adult beta cells exhibited an increased basal insulin secretion compared to P20 beta cells after preincubation at 5.6 mM glucose concentrations (Fig 5E). In addition, adult beta cells incubated at 5.6 mM glucose showed a higher insulin secretion upon stimulation with 15.6 mM glucose, compared to P20 beta cells. Furthermore, P20 beta cells preincubated with NGF at 5.6 mM glucose exhibited increased basal insulin secretion at this concentration than in control condition.

In contrast, adult beta cells preincubated at 15.6 mM glucose levels showed no increase in insulin secretion upon glucose stimulation with 15.6 mM glucose. P20 beta cells preincubated with NGF and 15.6 mM glucose did not exhibit an increase in insulin secretion upon stimulation with 15.6 glucose. Finally, neither P20 nor adult beta cells showed an increase in insulin secretion upon stimulation with 15.6 mM after preincubation with NGF at high glucose, or under high glucose conditions, respectively.

## Discussion

Our results show that variations in glucose concentrations induced changes in GLUT2 protein levels and subcellular distribution. After incubation at 15.6 mM glucose, adult rat beta cells exhibited an increase in GLUT2_T_ and GLUT2_PN_ compared to incubation at 5.6 mM glucose (Fig 1). Our results in adult beta cells confirm the results obtained in previous studies [30], which observed that various factors can influence the expression and cellular distribution of GLUT2. Elevated levels of extracellular glucose concentrations are known to increase GLUT2 expression and promote its translocation to the cell membrane and in adult beta cells increase the GSIS [29, 30]. However, incubation of adult beta cells with NGF at 15.6 mM glucose decreased levels of GLUT2_T_ and GLUT2_PN_ as detected by semiquantitative immunofluorescence, as well as glucose internalization at 15.6 mM glucose (Fig 3). Previous work has observed that long-term exposure of adult beta cells to high glucose levels could impair GLUT2 protein cellular localization and GSIS [31]. Furthermore, another study observed that GLUT2 expression and membrane localization decreased in beta cells after prolonged insulin secretion, suggesting that insulin secreted could exert negative feedback by inducing GLUT2 internalization [32]. The variations in GLUT2 expression levels in beta cells modulate the glycolytic flux and therefore the expression of genes involved in extracellular glucose sensing and glucose metabolism, such as insulin, GLUT2, GK and, some mitochondrial enzymes [7, 8].

On the other hand, incubation at 15.6 mM glucose decreases GLUT2_T_ and GLUT2_M_ levels in P20 beta cells. However, exposure to 5.6 mM induced an increase in GLUT2_T_ and GLUT2_M_ levels in beta cells at the same age. In addition, P20 beta cells showed higher levels of GLUT2_T_, GLUT2_M_, and GLUT2_PM_ than adult beta cells after incubation at 5.6 mM glucose (Fig 1). Although our results in P20 beta cells resemble previous observations made in beta cells obtained from hyperglycemic and hyperinsulinemic rats [31, 32], the differences in maturation stages, and assessment conditions (*in vitro*) hinder direct comparisons between the studies. To our knowledge, this is the first study to assess the effects of variations in glucose concentrations on the abundance and subcellular distribution of GLUT2 in pancreatic beta cells obtained from 20-day-old rats.

Moreover, our results showed that NGF increased GLUT2 total levels in adult rat pancreatic islets incubated at 5.6 mM of glucose. As previously mentioned, prolonged high-glucose stimulation and, thus, insulin secretion, result in a decrease in GLUT2 membrane expression due to its internalization leading to glucose uptake [32]. Short-term NGF effects enhance GSIS in pancreatic islets and isolated adult beta cells [33, 34]. Therefore, long-term incubation with NGF and 15.6 mM glucose may induce prolonged GSIS, leading to higher extracellular insulin levels, resulting in GLUT2 internalization and decreased glucose uptake. In contrast to its effects on adult beta cells, incubation with NGF at 5.6 mM glucose increased total protein levels of GLUT2 analyzed by immunoblot, as well as levels of GLUT2_T_ and GLUT2_PN_ observed by immunofluorescence in islets obtained from 20-day old rats (Fig 2).

Furthermore, incubation of P20 beta cells with NGF and 15.6 mM glucose increased GLUT2_M_ and GLUT2_PM_, and glucose uptake at 15.6 mM glucose conditions.

The increment of GLUT2 protein levels in P20 islets, and GLUT2_T_ and GLUT2_PN_ in P20 isolated beta cells reveals that NGF stimulation at 5.6 mM glucose may increase GLUT2 synthesis, leading to the protein accumulation near the nucleus. In addition, this could explain the lack of increase in glucose uptake at 5.6 mM concentrations in P20 beta cells after incubation with NGF. Moreover, NGF stimulation at 15.6 mM glucose did not increase protein levels in P20 islets. However, the increased levels of GLUT2_T_, GLUT2_M_, and GLUT2_PM_ in P20 beta cells after incubation with NGF at 15.6 mM could indicate an increase in GLUT2 vesicle translocation to the cell membrane, which may result in a higher glucose uptake observed at 15.6 mM glucose conditions (Fig 3).

It is well demonstrated that P20 beta cells show lower glucose uptake levels compared to adult beta cells (Fig 3), which correlates with the differences in GLUT2 protein levels observed between P20 and adult beta cells. Therefore, GLUT2 may play a crucial role by directly controlling glucose internalization across the cell membrane, and indirectly in the transcription of glucose-regulated genes, considering that both processes contribute to the development and maturation of beta cells [8, 35–37]. These results exhibit that subcellular distribution of GLUT2 varies among beta cells of different ages and is affected by glucose concentrations. Furthermore, NGF stimulates not only the synthesis but also GLUT2 translocation to the membrane in P20 pancreatic islet cells. These results suggest that NGF directly contribute to functional maturation of pancreatic beta cells by promoting the translation and vesicle translocation machinery; and indirectly by modulating transcription of glucose-regulated genes.

Our results show that P20 beta cells exhibit similar GK activity rate independently of glucose concentrations. On the other hand, adult beta cells showed an increased GK activity at 15.6 mM glucose when compared to 5.6 mM glucose conditions. These findings are similar to those obtained in other studies with adult beta cells, where GK activity rate increases at 10 mM glucose or higher concentrations and support that increases in glucose concentrations enhances its activity [38].

Glucokinase is the rate-limiting enzyme of intracellular glucose metabolism and plays a key role in GSIS, and variations in its activity could impair insulin secretion [39, 40]. Compared to other hexokinases, GK exhibits an optimal activity at approximately 7 mM glucose and lacks product-dependent inhibition by glucose-6-phosphate [41]. Variations in the activity-rate of GK depend on the conformational change between a closed form with low or null activity and an open form with high activity. Moreover, a super-open form with the highest activity has been described [42].

In pancreatic beta cells, regulation of GK by PFK-2/FBPase-2 and glucose has been demonstrated [14, 15]. Following cellular glucose internalization, glucose binds to GK and increases its activity rate and affinity, thus enhancing the binding of another glucose molecule. Conversely, when intracellular glucose concentrations decrease, GK shifts into a low-affinity and steady-state kinetic conformation [40, 43]. Our results confirm that GK activity is lower in immature P20 beta cells than in mature adult beta cells. Furthermore, our results suggest that immature beta cells lack the mechanisms to regulate GK conformational changes and activity in response to glucose.

In beta cells, GK may not only be found in the free form in the cytoplasm but also associated with the membrane of insulin vesicles or mitochondria [14, 41, 44]. Additionally, a relationship between the location and activity rate of GK has been suggested. GK displays a low or null activity rate when it is bound to insulin vesicles. However, after its release from vesicles to the cytoplasm, GK exhibits an open configuration with a high activity rate [45].

Moreover, no significant differences were observed in GK activity of P20 beta cells after incubation with NGF. However, NGF increased GK activity in adult beta cells at 15.6 mM compared to 5.6 mM glucose. The effects of NGF on the activity of GK in adult beta cells suggest that these cells differ from immature P20 beta cells in the regulatory mechanisms that control GK activity.

These results demonstrate that the activation GK at high glucose concentrations may constitute one of the mechanisms that develop during the maturation process from P20 to adult beta cells observed in previous studies [10]. Additionally, these findings may indicate that the regulation of insulin-vesicles traffic mediated by the TrkA-pathway differs between immature and mature beta cells. We hypothesize that this differential regulation could allow the release of GK from insulin vesicles to the cytoplasm and facilitate the acquisition of the open form of GK at high intracellular glucose concentrations. However, further research is needed to confirm our hypothesis.

Our findings demonstrate that not only P20 but also adult beta cells increased intracellular insulin levels after preincubation in 5.6 mM compared to 15.6 mM glucose. Additionally, adult beta cells preincubated at 15.6 mM glucose levels showed no increase in insulin secretion upon glucose stimulation with 15.6 mM glucose. Previous studies have suggested that the inhibitory effect on insulin secretion, resulting from prolonged incubation of beta cells to high glucose concentrations, may be related to the development of alterations in glucose metabolism or the degradation of the components of the exocytic machinery [32, 46]. In addition to insulin synthesis, the assembly and traffic of insulin secretory vesicles are regulated by glucose concentrations in a time-dependent manner [6, 32, 46, 47]. Other factors including hyperglycemia, increased insulin demand, and oxidative or endoplasmic reticulum stress have been proposed as inhibitors of insulin secretion [6, 47].

The lower levels of intracellular insulin after preincubation at 15.6 mM (4 hours) may partially explain the decreased GSIS of these cells at 15.6 mM of glucose observed in our analysis. Additionally, preincubation of adult beta cells with NGF and 15.6 mM glucose for 4 h (long-term) induces insulin secretion, which may be related to both the release of GK from insulin vesicles and its activation, as well as the depletion of the readily releasable insulin-vesicle pool. Moreover, the insulin secreted during the first hour of preincubation may exert negative feedback signaling resulting in GLUT2 internalization.

Furthermore, previous studies have demonstrated that after insulin secretion, its subsequent autocrine signaling also regulates GK localization and activity [40, 45]. Therefore, further investigation is needed to determine if NGF signaling could regulate GK translocation and activity, as well as its role in the modulation of GSIS of adult beta cells in the short- and long-term.

Additionally, our results show that preincubating P20 beta cells with NGF at 5.6 mM glucose increases GSIS after stimulation with 15.6 mM glucose without increasing glucose uptake. We propose that NGF effects on GLUT2 translocation and subcellular localization, as well as GSIS in P20 beta cells, depend on the maturity of the vesicular traffic machinery. In addition, the increase in GSIS after preincubation in these conditions suggests that NGF effects do not rely on glucose uptake. Finally, our findings reveal the presence of beta-cell subpopulations with different glucose uptake efficiency in 20-day- old and adult rats. These results agree with previous studies that describe beta-cell heterogeneity with differences in gene and protein expression, and in insulin secretion rate [13, 48, 49]. Our results show that preincubating P20 beta cells with NGF at 5.6 mM glucose increased the proportion of high-uptake (H) cells; thus, further analysis is needed to determine if this change also contributes to the increase observed in insulin secretion at 5.6 mM glucose conditions.

## Conclusion

Our work highlights that some differences between immature and mature beta cells are related to the subcellular distribution of GLUT2, and glucose uptake, and GK activity. We demonstrate that NGF could promote P20 beta cell maturation by inducing the subcellular redistribution of GLUT2, which results in an increase of GSIS.

## Supporting information

S1 Fig GLUT2-specific fluorescence levels in P20 pancreatic beta cells. (a) Control cells and cells incubated with NGF (50 ng/mL) for 5 minutes. (b) 1 hour. (c) 4 hours. Bars represent the mean ± S.E.M. * p <0.05, Student’s t test.
(TIF)

S1 Data (XLSX)

S2 Data (XLSX)

S1 Raw images (PDF)
