# Supplementary figures and images for: NGF effects promote the maturation of rat pancreatic beta cells by regulating GLUT2 levels and distribution, and glucokinase activity
Source: PLoS One. 2024 Jun 14;19(6):e0303934. doi: 10.1371/journal.pone.0303934 (PMC11178159; doi:10.1371/journal.pone.0303934)

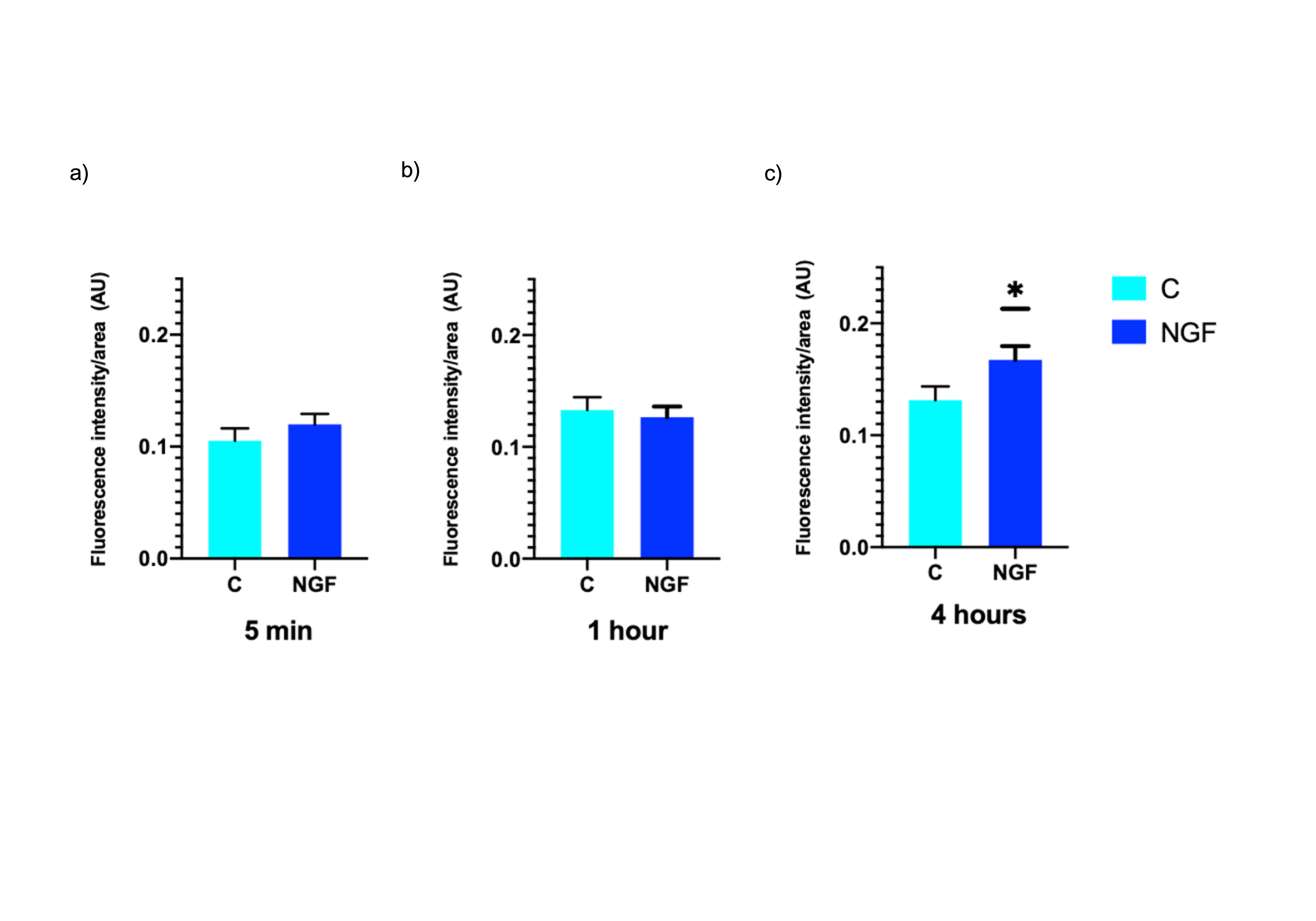

Supplement: S1 Fig — (a) Control cells and cells incubated with NGF (50 ng/mL) for 5 minutes. (b) 1 hour. (c) 4 hours. Bars represent the mean ± S.E.M. * p <0.05, Student’s t test. (TIF) [file pone.0303934.s001.tif]
