# Supplementary material for: NGF effects promote the maturation of rat pancreatic beta cells by regulating GLUT2 levels and distribution, and glucokinase activity
Source: PLoS One. 2024 Jun 14;19(6):e0303934. doi: 10.1371/journal.pone.0303934 (PMC11178159; doi:10.1371/journal.pone.0303934)
Supplement: S1 Raw images — (PDF) [file pone.0303934.s004.pdf]

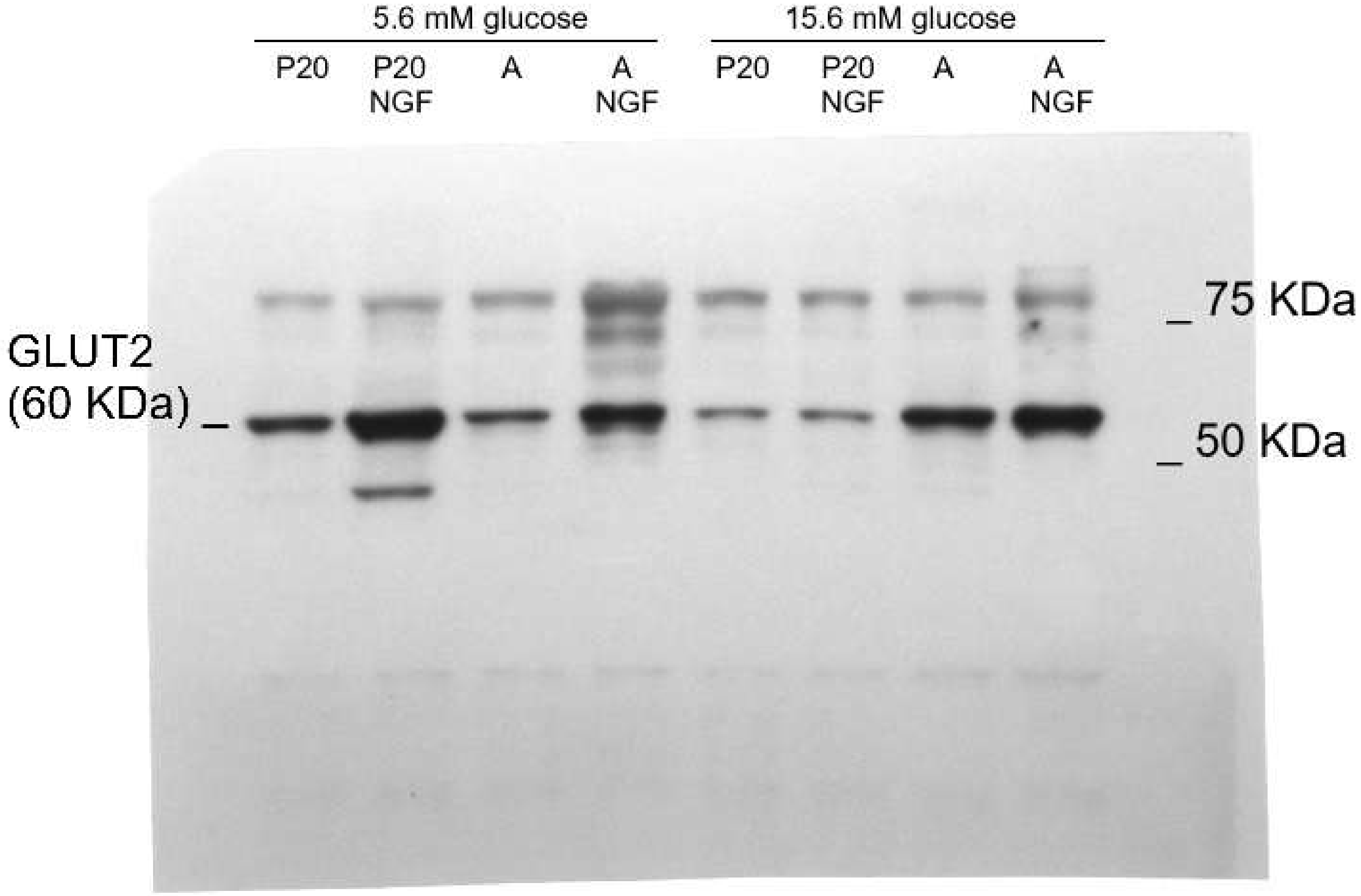
5.6 mM glucose
15.6 mM glucose
P20
P20 NGF
A
A NGF
P20
P20 NGF
A
A NGF
GLUT2 (60 KDa)
75 KDa
50 KDa

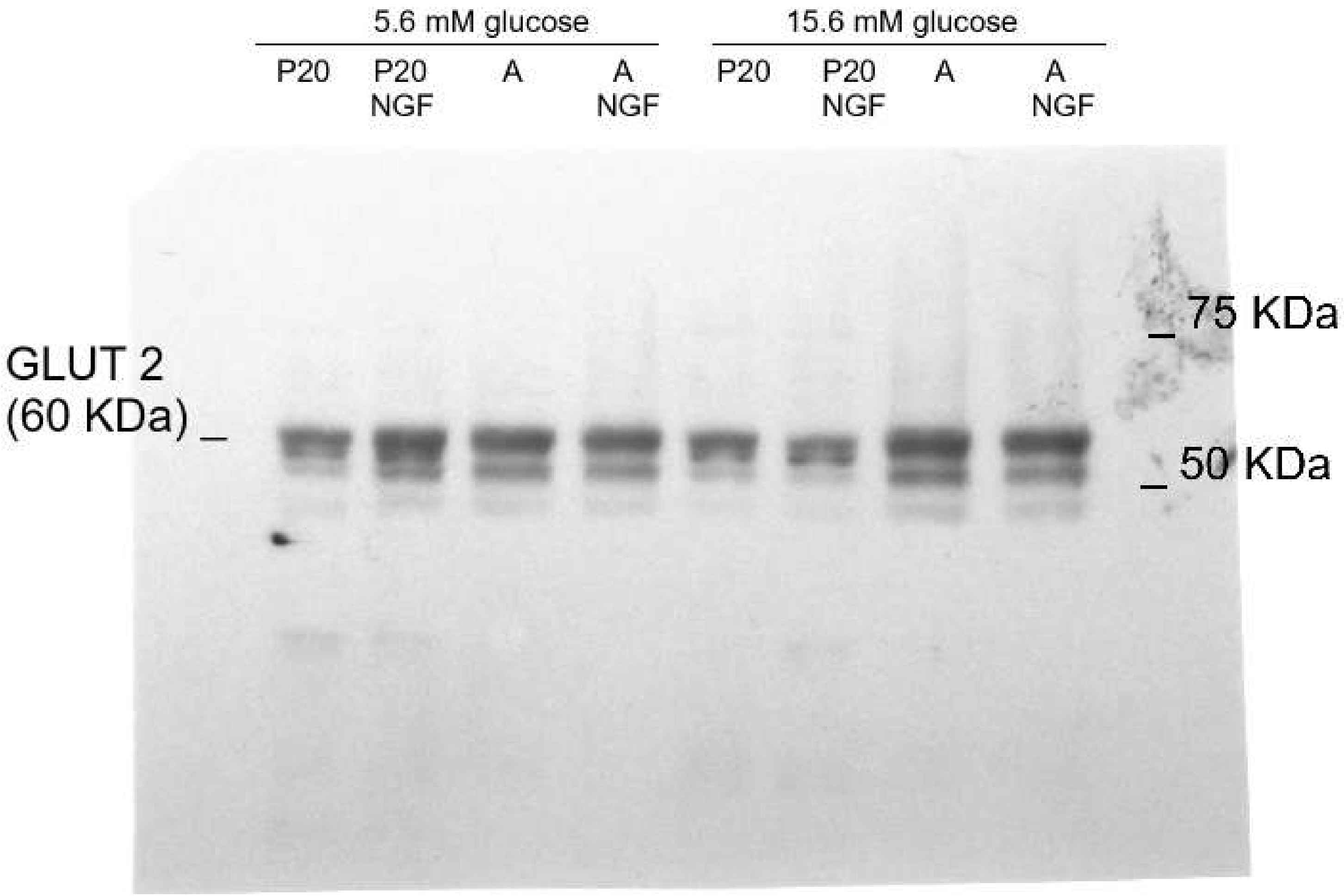
5.6 mM glucose
15.6 mM glucose
P20
P20 NGF
A
A NGF
P20
P20 NGF
A
A NGF
GLUT 2 (60 KDa)
75 KDa
50 KDa

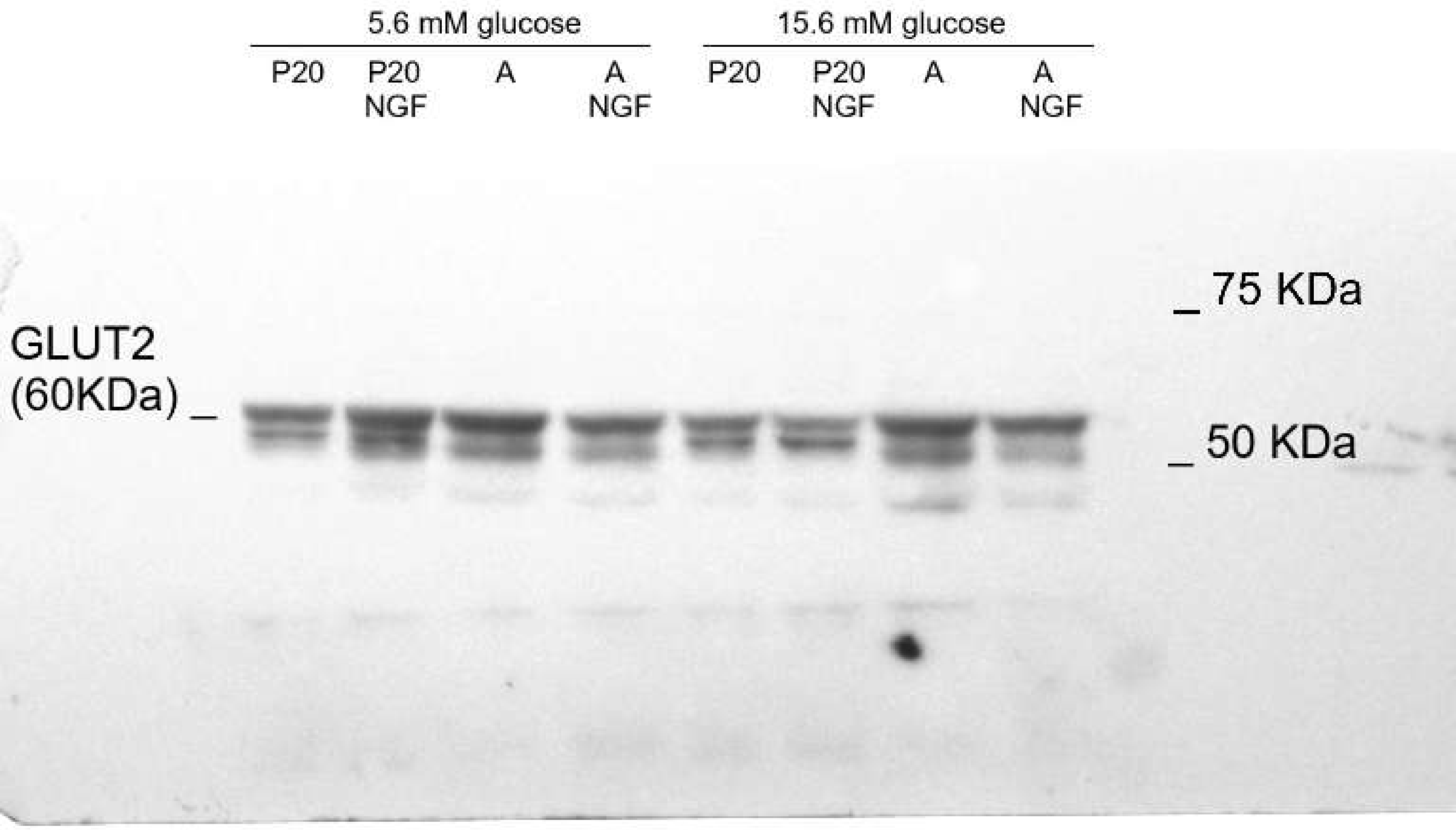
5.6 mM glucose
15.6 mM glucose
P20
P20 NGF
A
A NGF
P20
P20 NGF
A
A NGF
75 KDa
GLUT2 (60KDa)
50 KDa

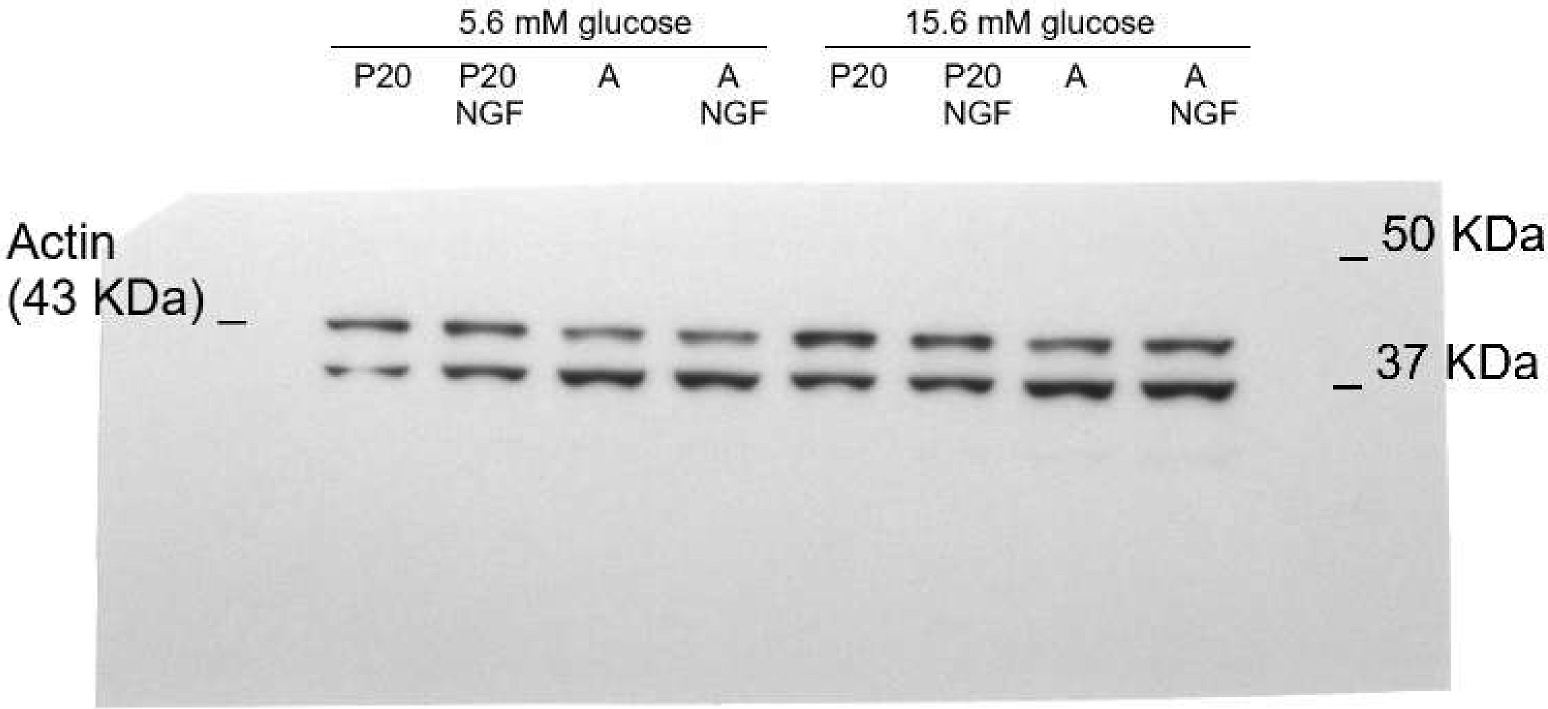
5.6 mM glucose
15.6 mM glucose
P20
P20 NGF
A
A NGF
P20
P20 NGF
A
A NGF
Actin (43 KDa)
50 KDa
37 KDa

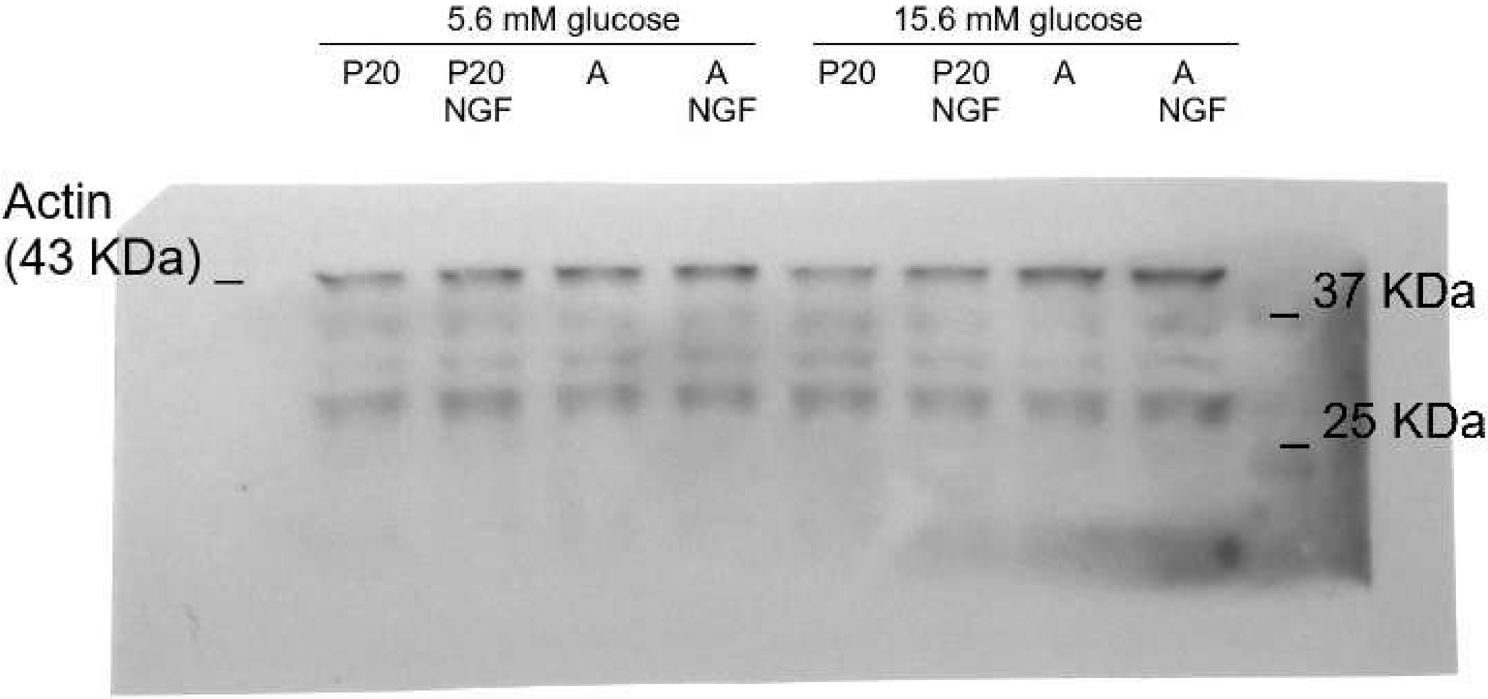
5.6 mM glucose
15.6 mM glucose
P20
P20 NGF
A
A NGF
P20
P20 NGF
A
A NGF
Actin (43 KDa)
37 KDa
25 KDa

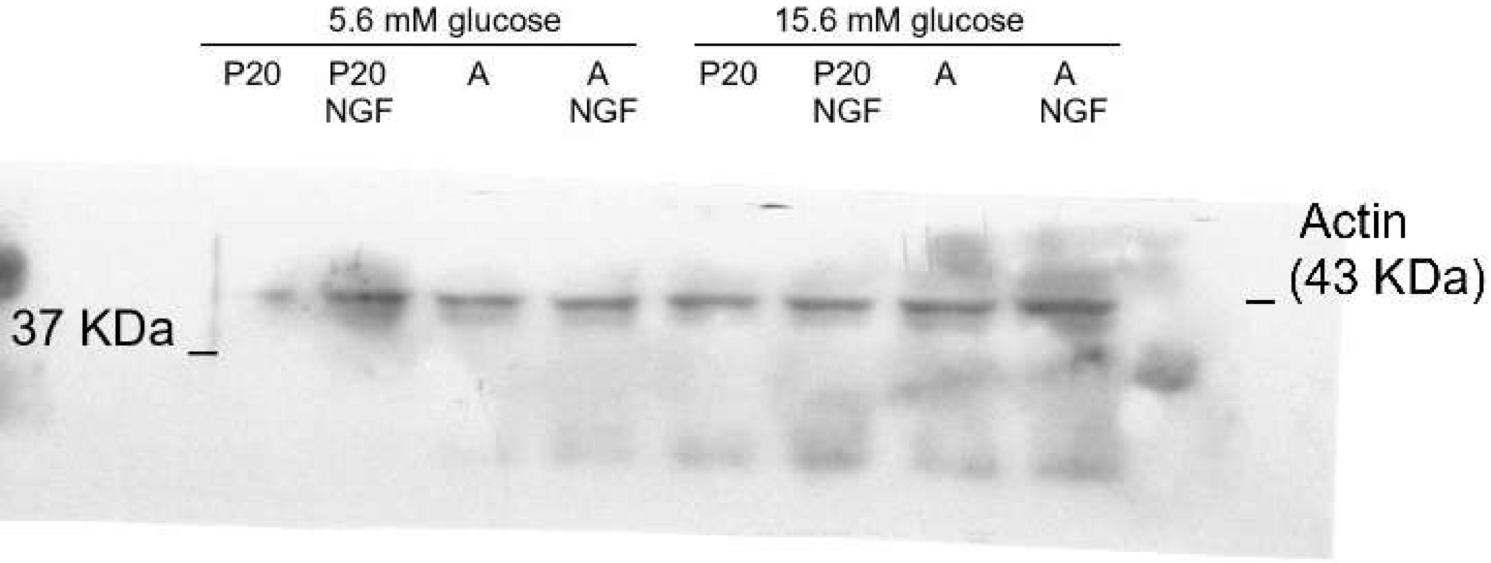

Proteins were visualized using a chemiluminescent HRP substrate method with Immobilon Western Chemiluminescent HRP subtrate (#WBKLSO100, Merck Millipore, Mass, USA) on KODAK photofilms. Image scanning was done with epson multifunctional scanner L375.
